# What can case-control comparisons of neural drug cue reactivity tell us about addiction? A neuroimaging meta-analysis

**DOI:** 10.1038/s41398-026-04458-9

**Published:** 2026-09-17

**Authors:** Maximilian Fascher, Krzysztof Krasowski, Markus Muehlhan

**Affiliations:** 1 Department of Psychology, Faculty of Human Sciences, Medical School Hamburg, Am Kaiserkai 1, 20457 Hamburg, Germany; 2 ICAN Institute for Cognitive and Affective Neuroscience, Medical School Hamburg, Am Kaiserkai 1, 20457 Hamburg, Germany

**Keywords:** Addiction, Diagnostic markers, Prognostic markers, Neuroscience

## Abstract

Neural drug cue reactivity (DCR) is a central paradigm in addiction neuroscience for probing how drug cues acquire excessive motivational salience in substance use disorders (SUDs). However, the field faces challenges in clinical translation, replicability, and within-group designs are susceptible to visual confounding due to stimulus matching. A complementary approach is to examine case-control contrasts to identify SUD-specific neural architecture while mitigating effects of task-design variability. We conducted a systematic literature search and an activation likelihood estimation (ALE) meta-analysis to identify convergence effects in the contrast drug > control cues between SUD and control groups. ALE results were supplemented by assessing behavioural domains, meta-analytic connectivity profiles, robustness to publication bias, and study-level modulators using penalised logistic regression. The ALE analysis revealed one reliable convergence cluster in the ventral anterior cingulate cortex (vACC), associated with reward, reasoning, and social cognition. Penalised logistic regression indicated that contributions to this cluster were less likely in alcohol DCR-studies and were sensitive to matching conditions for human content. vACC DCR effects contrasted with neutral control cues were invariant across abstinence periods, whereas vACC DCR contrasted with natural reward cues declined with longer abstinence. This meta-analysis replicates context-sensitive SUD-specific DCR convergence in vACC regions intersecting reward pathways and the default-mode network, while reliable striatal convergence was not observed. The review recapitulates these results by discussing current challenges in balancing personalisation and standardisation, the use of motivationally inert versus salient comparators, and argues for careful deliberation when inferring evidence for hyperreactive reward responses.

## Introduction

Substance use disorders (SUDs) are severe mental health conditions that profoundly compromise physical and psychological well-being while placing substantial burdens on families, communities and healthcare systems [1]. A central feature of SUDs is a progressive loss of control over drug intake [2], in which individuals continue to use substances despite experiencing significant and often worsening harm to their health, social relationships, and overall quality of life [3]. To many people without SUD, the rapid recurrence and persistence of drug use despite serious consequences may seem paradoxical. However, relapse remains a defining and frequent element that shapes the lived experience of those affected by SUD. Therefore, gaining a better understanding of the factors that promote the development and persistence of SUD is a crucial goal in psychiatric research.

One way to approach the complexity of the disorder is to examine its neural underpinnings using functional magnetic resonance imaging (fMRI). Neural drug cue reactivity (DCR) has become an influential paradigm, particularly in fMRI research, where it is widely used to probe cue-triggered neural processes associated with craving and motivational salience in individuals with SUD [4–7]. This framework has often been used to link SUD neurobiology to influential theoretical accounts, such as incentive sensitisation theory [8, 9] and the impaired response inhibition and salience attribution model of drug addiction (iRISA [10]). Both theories propose that drugs acquire abnormally strong motivational salience, exceeding that elicited by natural reinforcers, which is reflected in exaggerated mesolimbic or cortico-striatal responsivity.

The neural architecture underlying DCR is increasingly well characterised, as numerous neuroimaging studies and meta-analyses have shown that DCR recruits a widely distributed array of regions, including midline hubs of the default mode network (DMN), as well as striatal, temporo-occipital, parietal, and limbic areas. This widespread activation reflects the involvement of diverse cognitive and affective processes [11–15]. Recently, a neuroimaging meta-analysis by our group demonstrated that dynamics in mesocorticolimbic pathways and the DMN vary as a function of clinical severity [13], underscoring the potential of DCR to link neural activity to SUD-relevant clinical metrics. Similarly, neural DCR in regions such as the nucleus accumbens have been shown to predict relapse in SUD [16, 17]. There is also evidence that nucleus accumbens DCR may be more effective in predicting relapse occurrence than characteristics captured by self-report assessments or clinician-guided interviews [18].

At the same time, considerable methodological and clinical heterogeneity, limited translation from neural measures to behaviour and clinical outcomes [19] and the sheer volume of available data have prompted large collaborative initiatives [20]. These efforts aim to advance biomarker development for addiction by leveraging this extensive body of literature [6]. For any such translational effort, however, it may be essential to consider the specific context of use for which a biomarker is to be developed. In this regard, neural DCR may inform developments in diagnostic, prognostic, response, predictive, monitoring or susceptibility related biomarkers of SUD, each with potentially distinct implications for research and inventing novel treatments [6]. While the interaction contrast comparing drug > control cues in individuals with substance use disorders relative to non-affected controls most directly captures disorder-level differences, DCR-derived biomarkers are unlikely to surpass established clinical assessments in terms of practicality, cost efficiency, or feasibility for diagnostic purposes [6]. Nonetheless, when incorporated into a dedicated neuroimaging meta-analysis, the cue-by-group interaction offers two important advantages that extend beyond within-subject DCR contrasts.

First, it addresses a distinct translational question by identifying neural responses to drug cues that differentiate individuals with SUD from non-affected controls, a distinction that cannot be established from within-subject contrasts alone. Second, the interaction contrast enables a more specific characterisation of cue-evoked activity by separating substance-related neural responses from activity driven by general perceptual, attentional, or task-inherent processes. When drug and control cues are insufficiently matched, within-subject contrasts may conflate substance-specific effects with variance introduced by stimulus or task properties. This concern is not merely theoretical: recent meta-analytic work demonstrates that many DCR paradigms insufficiently control for low-level perceptual features involving action observation or human stimulus content, which can inflate neural responses in visual association cortices [13]. Such design heterogeneity is especially consequential for meta-analyses, where task-level differences can aggregate into convergence patterns that are not theoretically informative. Given that DCR paradigms reliably engage distributed neural systems implicated in reward-related, executive, and sensory functions, within-subject contrasts alone may be insufficient to determine whether activity in these regions reflects substance-specific cue processing in SUD or more general stimulus-driven neural responses. By directly comparing SUD and control participants viewing the same stimuli in each experimental set-up, these confounds are mitigated, allowing the isolation of neural effects that are more likely to reflect SUD-related processing of drug cues.

However, although there is growing recognition that the neural circuits engaged by DCR substantially overlap with those involved in natural reward processing [11, 12], this gap in the empirical literature remains largely unaddressed, as previous neuroimaging meta-analyses including our own work have primarily focused on within-subject designs [13–15], have been restricted to individual substance classes such as nicotine [16] or alcohol [21], or have merged within-subject and between-group DCR contrasts within a single synthesis [11, 12]. To our knowledge, the present study is the first neuroimaging meta-analysis to specifically examine this drug cue by group interaction contrast. We applied the Activation Likelihood Estimation algorithm [22] to quantify above chance convergence across studies and conducted a set of standard follow-up analyses including characterisation of task-related behavioural domains [23], meta-analytic task-based connectivity profiles [24, 25] and an assessment of robustness to potential publication bias using a Fail-Safe-N approach tailored to ALE [26]. Finally, we used a penalised logistic regression approach to examine whether study level variables, including design and clinical features, predict the likelihood of observing a given cluster in a specific study. This exploratory analysis builds on our previous meta-analysis of the within-SUD contrast [13], which showed that DCR is systematically modulated by clinical and methodological factors, and extends this approach to assess whether similar modulations emerge for the interaction contrast comparing individuals with SUD to non-affected controls.

## Methods

### Systematic literature search

This systematic literature review was pre-registered by our research group as part of a previous meta-analysis that examined the within-subject design of drug > control stimuli in substance users [13] (https://www.crd.york.ac.uk/PROSPERO/view/CRD42024556138). We used EBSCOhost (https://search.ebscohost.com/) including the databases PsycINFO, PsycARTICLES, Medline Complete, CINAHL Complete, and the Psychology and Behavioral Sciences Collection and expanded the study pool with an equivalent search in PubMed. The literature search was conducted by researcher MF. All studies that passed the title and abstract screening were reviewed by researcher MF and KK for compliance with the inclusion criteria. In case of disagreement between the reviewers, researcher MM was consulted after discussion. We documented the adherence to PRISMA-guidelines for systematic reviews [27] and guidelines for neuroimaging meta-analyses [28] in Supplementary Tables S1 and S2. In direct reference to our previous meta-analysis [13] our inclusion and exclusion criteria were as follows:

(1) We only considered whole-brain and almost whole-brain studies to be eligible as this is a prerequisite for statistical assumptions of the ALE algorithm [22, 28]. Accordingly, region-of-interest (ROI) and small-volume corrected results were excluded. (2) To minimise inflation introduced by false-positive findings and data noise, coordinates from whole-brain case-control contrasts were eligible when source studies reported either formal correction for multiple comparisons or an explicitly specified cluster-extent thresholding procedure. (3) All functional brain findings had to be reported in a standardised reference space, i.e. either Talairach or Montreal Neurological Institute (MNI). (4) We required that all studies examined at least 10 subjects in the SUD group to achieve a minimum statistical power per included contrast. (5) SUD samples had to be at least 14 years old on average acknowledging almost adult cranial size [29] should not introduce major bias due to normalisation procedures. (6) Control participants were required to not meet SUD-criteria for the target substance under investigation. All eligible studies were checked for potential sample overlap and, if detected, only the study with the larger sample size was included. Integrating overlapping samples as independent in the analysis leads to an overestimation of the replicability of neuroimaging findings [28]. We applied the same search string we used in our previous meta-analysis [13]:

(“cue reactivity” OR “cue-reactivity” OR “cue exposure” OR “cue-exposure” OR “cue-elicited craving” OR “drug cue reactivity” OR “drug cue-reactivity” OR “drug cue exposure” OR “drug-cue exposure” OR “drug cue-elicited craving”) AND (“functional magnetic resonance imaging” OR “fMRI” OR “functional MRI”) AND (“substance use disorder” OR “addiction” OR “drug abuse” OR “drug dependence” OR “alcohol use disorder” OR “alcoholism” OR “alcohol” OR “drinker*” OR “cocaine” OR “crack” OR “methamphetamine” OR “crystal meth” OR “amphetamine” OR “stimulant*” OR “tobacco” OR “smok*” OR “cannabis” OR “THC” OR “opioid*” OR “heroin” OR “morphine” OR “codeine” OR “ketamine” OR “polytox*” OR “polysubstance” OR “substance use*”).

### Activation likelihood estimation and consecutive cluster assessments

In this paper we used Activation Likelihood Estimation (ALE [22, 30]) as the coordinate-based meta-analytic algorithm to synthesise the fMRI findings of neural DCR in SUD compared to non-affected controls (drug > control × SUD > controls) as noted above. ALE determines above-chance convergence of neuroimaging study effects across integrated studies by accounting for the spatial uncertainty innate to fMRI data being highly processed in general and subject to various unique preprocessing pipelines and cross-sample variability. Thus, ALE models a 3D Gaussian probability function of activation likelihood centred around statistical cluster peaks (i.e., foci) reported across synthesised studies. Instead of assigning weight to individual studies based on samples size, larger samples contribute with a higher spatial precision (i.e., denser probability function kernels) than smaller samples do [30, 31]. Taking together all modelled voxel-wise probability functions results in a modelled activation map per study and their aggregation finalises into an ALE-map denoting an ALE estimate of spatial convergence across integrated experiments. Accordingly, the main estimators of effect are convergence values (i.e., ALE-scores), cluster extents and the number of studies contributing to an ALE-derived cluster. To test whether aggregated ALE maps show regional convergence against a just random spatial distribution of foci that could be detected by chance we applied 1000 permutation tests of random spatial allocations of the foci integrated. All ALE analyses underly a state-of-the-art multiple comparison correction for ALE meta-analyses by applying the voxel-wise threshold of p < 0.001 and the cluster-forming threshold of p < 0.05 of family-wise error correction (cFWE) [28]. Preceding the ALE meta-analysis, we corrected 4 foci that appeared to be reported incorrectly due to sign errors. See also Supplementary Table S3 for the foci corrected before entering the ALE meta-analysis. Further, all foci that were reported in Talairach reference space have been transformed to MNI reference space using the Lancaster transform (icbm2tal [32]) implemented in the GingerALE software. This way we minimised transformation costs as most experiments reported their foci in MNI reference space.

We then employed a standard pipeline of consecutive analyses of ALE derived cluster assessments, which is described in greater detail in the Supplementary Methods. In brief, we first conducted a behavioural domain analysis (BDA [23]) using the BrainMap database (https://brainmap.org) of functional domains to specify a task-based behavioural profile for ALE-derived clusters. We then performed a meta-analytic connectivity modelling (MACM [24, 25]) to characterise their task-positive meta-analytic connectivity profiles and to delineate the large-scale network architecture in which the ALE clusters participate. Finally, we evaluated the robustness of each cluster to potential publication bias by conducting a Fail-Safe-N (FSN) analysis tailored to ALE results [26]. In this procedure, artificial noise is iteratively introduced into the dataset to determine whether a cluster maintains relative stability facing additional hypothetical null findings or whether its apparent significance is disproportionately shaped by a small subset of unduly influential studies.

### Linking ALE cluster contributions to clinical variables

Because the number of available studies did not provide the required power for meta-analytic contrast and conjunction analysis [24] to investigate the modulatory properties of study level characteristics on above-chance convergence, we adopted an alternative strategy. In its place, we examined whether the likelihood that a study contributed to any ALE-derived cluster was associated with variations in study level variables. For this purpose, we used penalised logistic regression with Firth correction [33] implemented in JASP [34] (version 0.19.3.0). This approach reduces bias in maximum likelihood estimation by modifying the likelihood function and is particularly suitable for small samples, situations of full separation in which one factor level contains only “zeros”, and strongly unbalanced designs [35, 36]. In these analyses, the binary dependent variable indicated whether a study contributed to a given cluster, coded as contribution or non-contribution. The predictors were a set of design-related and clinical variables that were selected in accordance with our recent meta-analytic synthesis [13].

Design-related variables included the valence of the control condition, classified as neutral or appetitive, and the degree to which drug and control stimuli were matched with respect to human-related and action- or tool-related content. A condition was classified as non-matched when drug cues contained human, action, or tool features that were absent in the corresponding control cues.

Clinical variables included substance class, mean duration of substance use, abstinence duration, and treatment-seeking status. Substance classes were pragmatically grouped into alcohol, nicotine, and a heterogeneous category comprising other substances such as heroin, methamphetamine, and cannabis. This approach was adopted to limit the number of regressors to a feasible minimum while accommodating the disproportionate representation of alcohol and nicotine in the literature relative to less frequently studied substances. For abstinence duration, we extracted the reported mean whenever available. When studies reported only a range, abstinence duration was approximated using the midpoint of the reported interval. In cases where abstinence was reported using lower-bound descriptors (e.g., “at least two hours”), the lower boundary was used as a conservative estimate reflecting the minimal abstinence duration implied by the reporting format. Treatment-seeking status was defined based on whether participants were described as seeking or currently receiving treatment, as opposed to samples without such characterisation. With this definition we aimed at capturing a motivational tendency for participants to quit drug consumption.

Given the exploratory nature of the analyses, the modest number of available studies, and heterogeneity in reporting across the literature, study-level predictors were evaluated using a series of separate Firth’s penalised logistic regression models. Each model included a single predictor or one two-way interaction, and no model contained more than two predictors, in order to minimise the risk of overfitting under conditions of limited sample size. Studies lacking information on a given characteristic were excluded only from analyses involving that specific predictor. Prior to model fitting, the distributions of all predictors were inspected. Abstinence duration was log-transformed due to its highly skewed and bimodal distribution, spanning from hours to several hundred days. To reduce the number of formal statistical tests, predictors were initially examined using descriptive visualisations of main effects and pairwise interactions. Only predictors for which these exploratory inspections suggested a potentially meaningful association were subsequently subjected to formal inferential testing. Statistical significance was defined as α ≤ 0.05 for both overall model fit and individual predictors. Model and predictor significance were evaluated using deviance-based likelihood ratio tests (db-LRT), with effect sizes reported as penalisation-adjusted odds ratios (aORs) and corresponding 95% confidence intervals.

## Results

### Study characteristics and literature search results

Our systematic literature search finalised on 23 eligible studies [16, 17, 37–57] comprising 25 independent contrasts, encompassing a total of N = 794 participants with SUD that we included in our main ALE meta-analysis. The PRISMA flow-chart is provided in Fig. S1 of the Supplementary Materials, and Table 1 summarises the demographic, clinical, and experimental characteristics of the included experiments.

**Table 1:** Overview of included studies synthesised in the ALE meta-analysis.

Sample #	Publication	Diagnostic assessment	Substance	SUD N	♀ %	SUD age	SUD duration (y)	Abstinence (days)	Treatment seeking	Contrast(s)	Thresholding	Foci
1	Bach et al. (2019)	DSM-IV alcohol dependence	alcohol	50	0.0	45.6 ± 8.9	13.1 ± 10.1	23.2 ± 15.2	yes	drug > neutral	voxel p < 0.001 cluster FWE p < 0.05	5
2	Beck et al. (2018)	DSM-IV and ICD-10 alcohol dependence	alcohol	23	30.4	46.2 ± 6.2	13.3 ± 9.5	12.3 ± 4.12	yes	drug > appetitive	voxel p < 0.001 cluster k > 5	1
3	Brumback et al. (2015)^**a**^	>100 lifetime drinking episodes, ≥3 past month heavy episodic drinking occasions, ≥1 withdrawal symptom	alcohol	22	55.0	17.9 ± 0.7	n.r.	n.r.	no	drug > appetitive	cluster k > 30, p < 0.01	3
4	Dakhili et al. (2022)	DSM-IV methamphetamine dependence	methamphetamine	53	0.0	32.1 ± 5.9	12.2 ± 6.4	81.6 ± 121.5	yes	drug > neutral	voxel Z > 3.1 cluster p < 0.05	4
5	Filbey et al. (2016)^**a**^	5000 lifetime occasions, daily use in last 60 days	cannabis	53	37.7	30.7 ± 7.5	12.2 ± 6.4	3	no	1. drug > neutral 2. drug > appetitive	voxel Z > 2.3 cluster p < 0.05	40
6	Fukushima et al. (2020)	DSM-5 alcohol use disorder	alcohol	24	29.0	47.5 ± 8.6	8.7 ± 6.9	30–60	yes	drug > appetitive	voxel p < 0.001 cluster FWE p < 0.05	1
7	Goudriaan et al. (2010)	≥15 cigarettes per day^**b**^	nicotine	10	0.0	33.8 ± 9.1	n.r.	0.25–0.75	yes	drug > neutral	cluster FWE p < 0.05	7
8	Goudriaan et al. (2013)	DSM-IV cocaine dependence	cocaine	13	n.r.	37.7 ± 9.5	n.r.	> 21	yes	drug > neutral	voxel p < 0.001 cluster k > 30	6
9	Heinz et al. (2007)	DSM-IV and ICD-10 alcohol dependence	alcohol	12	50.0	39.0 ± 7.0	n.r.	14 ± 4.2	yes	drug > neutral	voxel p < 0.001 cluster k > 5	6
10	Huang et al. (2018)^**a**^	DSM-IV methamphetamine dependence	methamphetamine	28	0.0	31.7 ± 7.1	5 ± 2.8	555 ± 79.2	yes	drug > baseline	cluster FWE p < 0.05	2
11	Huang et al. (2024)	DSM-5 opioid use disorder	heroin	32	21.9	40.3 ± 8.8	11 ± 6.7	196.8 ± 255	yes	1. drug > neutral 2. drug > appetitive	voxel z > 3.1, cluster FWE p < 0.05	12
12	Ihssen et al. (2011)^**a**^	British Medical Association hazardous and harmful drinkers	alcohol	11	9.1	26.9 ± 6.3	n.r.	n.r.	n.r.	drug > neutral	cluster k > 837	2
13	Kaag et al. (2018)	DSM-IV cocaine abuse or dependence	cocaine	59	0.0	31.4 ± 7.6	6 ± 12	n.r.	no	drug > neutral	voxel p < 0.001 cluster FWE p < 0.05	13
14	Kroon et al. (2024)	DSM-5 cannabis use disorder	cannabis	104	44.0	23.0 ± 2.0^**c**^	4 ± 2^**c**^	mostly < 1	no	1. drug > neutral 2. drug > neutral + appetitive	voxel Z > 3.1 cluster p < 0.05	12
15	Kunas et al. (2021)	DSM-5-TR tobacco use disorder	nicotine	38	55.6	35.2 ± 10.6	14.9 ± 10.6	0.125	yes	drug > appetitive	cluster FWE p < 0.05	1
16	Li et al. (2014)	DSM-IV heroin dependence	heroin	44	0.0	35.0 ± 7.1	6.7 ± 5.8	657 ± 432	yes	drug > baseline	cluster p < 0.05 (AlphaSim)	33
17	Liao et al. (2018)^**a**^	DSM-IV ketamine dependence	ketamine	40	20.0	26.8 ± 4.9	3.5 ± 1.8	> 0.083	yes	drug > baseline	cluster p < 0.005 (AlphaSim)	8
18	MacNiven et al. (2018)^**a**^	DSM-5 stimulant use disorder	stimulants	36	5.6	43.4 ± 13.3	17.6 ± 12.7	61.4 ± 10.4	yes	1. drug > neutral 2. drug > appetitive	voxel p < 0.001 cluster p < 0.05	2
19	Sjoerds et al. (2014)	DSM-IV alcohol dependence	alcohol	30	46.7	46.5 ± 8.5	15 ± 11.8	12.2 ± 20.4	yes	drug > neutral	voxel p < 0.005 cluster k > 15	7
20	Tabatabaei-Jafari et al. (2014) (1)	DSM-IV heroin dependence	heroin	20	0	32.0 ± 4.4	11.4 ± 4.6	276 ± 168	yes	drug > neutral	voxel Z > 2 cluster p < 0.05	4
21	Tabatabaei-Jafari et al. (2014) (2)			20		33.6 ± 4.7	11 ± 8.2	368 ± 297	yes			4
22	Zanchi et al. (2015) (1)	≥10 cigarettes for ≥2 years^**b**^	nicotine	14	71.4	29.3 ± 6.0	8.6 ± 4.5	0.01	no	drug > neutral	cluster TFCE p < 0.05	2
23	Zanchi et al. (2015) (2)			18		30.5 ± 5.7	8.1 ± 4.9	1350.5 ± 1095	yes			3
24	Zhang et al. (2011)^**a**^	‘smokers’^**b**^	nicotine	22	50	31.0 ± 9.4	12 ± 7.3	> 0.083	n.r.	drug > neutral	cluster FWE p < 0.05	6
25	Zhou et al. (2019)	DSM-IV cannabis dependence	cannabis	18	0	22.9 ± 2.7	5.2 ± 2.9	1	n.r.	drug > neutral	voxel p < 0.001 cluster FWE p < 0.05	25

*DSM* Diagnostic and Statistical Manual of Mental Disorders, *FWE* family-wise error correction, *ICD* International Statistical Classification of Diseases and Related Health Problems, *n.r*. not reported, *SUD* Substance use disorder, *TFCE* threshold-free cluster enhancement.

^**a**^The study is excluded in the sensitivity analysis.

^**b**^Although the study did not report minimum inclusion criteria thresholds for an SUD diagnosis, the means, standard deviations and ranges reported for the metrics ‘cigarettes per day’ and ‘cigarette use years’ imply chronic tobacco usage.

^**c**^Values refer to a median and median absolute deviation (MAD).

Methodological assessments and subsequent consensus discussions among MF, KK, and MM motivated performing a sensitivity analysis in which seven studies [17, 38, 42, 47, 48, 52, 56] were omitted from the main ALE due to methodological or diagnostic caveats (e.g., issues in fMRI contrast modelling or insufficiently specified SUD diagnoses) that, however, did not directly violate our pre-defined inclusion criteria. A detailed account of these studies and the rationale for them to be omitted in sensitivity analysis testing is provided in Supplementary Table S4. This step was undertaken to evaluate whether methodological caveats regarding data integrity systematically altered the ALE inferences. Excluding these studies resulted in a sensitivity-analysis sample of 16 studies with 18 independent sample contrasts, comprising N = 588 SUD participants.

### ALE meta-analysis results and robustness assessments

**Fig. 1:**
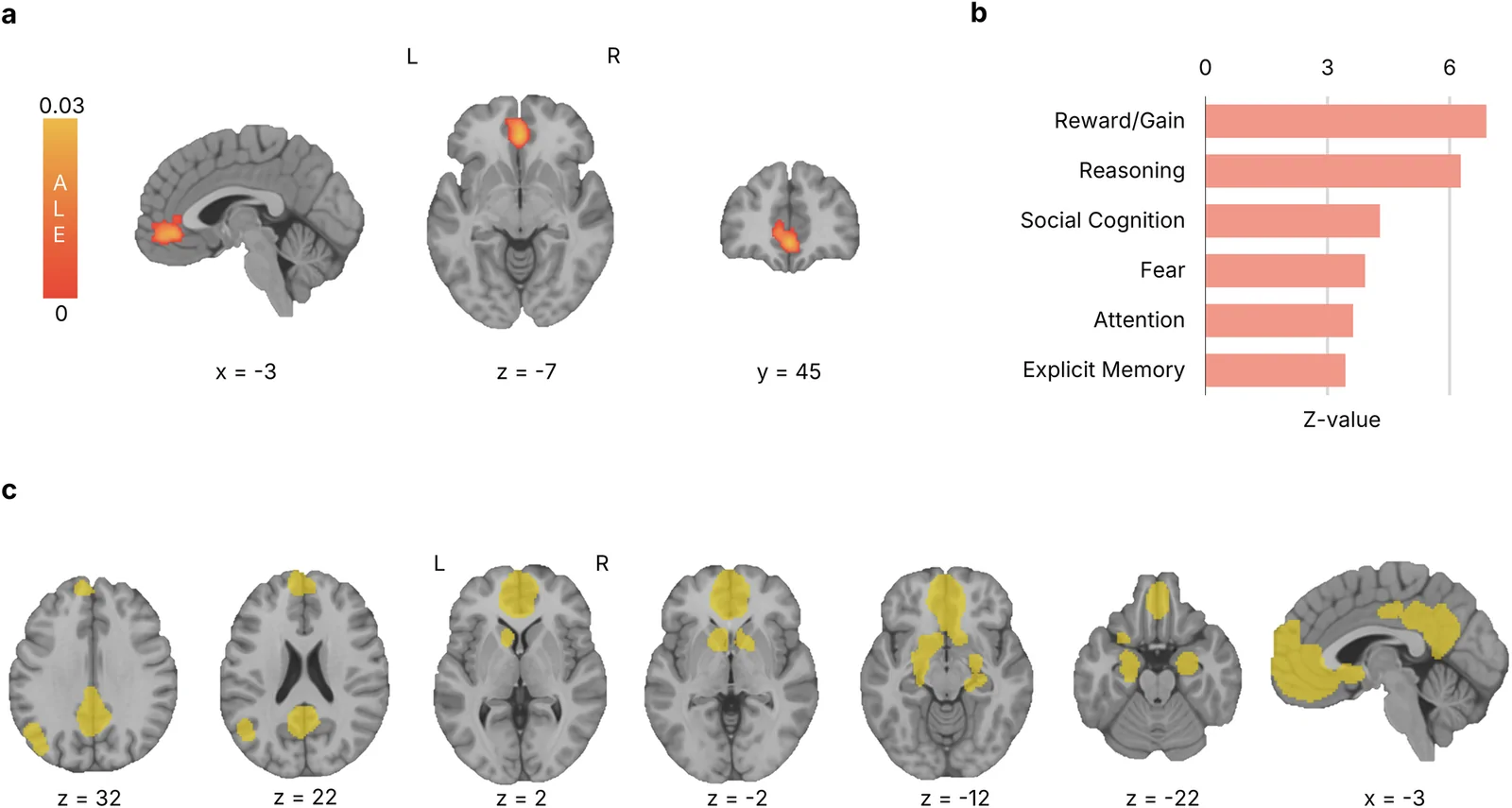
Visualisation of the ALE results, behavioural characterisation and meta-analytic connectivity modelling. Displayed are the ALE-derived cluster in the ventral anterior cingulate cortex (p < 0.001, cFWE p < 0.05, 1000 permutations) (**a**), the significant behavioural domains surpassing the threshold of Z = 3 the cluster is associated with (**b**) and its meta-analytic connectivity modelling (p < 0.001, cFWE p < 0.05, 1000 permutations) (**c**). *ALE* Activation Likelihood Estimation.

The main ALE meta-analysis showed one significant cluster of above-chance convergence peaking in a ventral division of the anterior cingulate cortex (vACC) for the comparison drug > control cues in SUD participants > control participants (Table 2, Fig. 1a). Regarding our sensitivity analysis assessments we tested whether the likelihood to contribute to this cluster was modulated by whether a study raised a methodological caveat or not. There was no difference in vACC contribution likelihood between the 7 studies being discarded in the sensitivity analysis due to methodological caveats and the remaining 16 studies (db-LRT: χ²(1) = 0.86, p = 0.354), demonstrating that the 7 studies omitted in the sensitivity analysis were not more or less likely to report an effect in vACC regions. Then, by repeating the ALE meta-analysis without the aforementioned studies, we also found this cluster in our sensitivity analysis ALE to be significant (Table S5, Fig. S2) with a similar ratio of studies contributing to this cluster (~44%), although its extent and maximum ALE values of peaking coordinates decreased expectedly.

**Table 2:** Main ALE results. Results are shown for the contrast drug > control in SUD > control participants (k = 25), using a cluster-forming threshold of p < 0.001 and cluster-level FWE correction at p < 0.05 with 1000 permutations.

				MNI (peak voxel)					
#	Anatomical peak region	mm³	BA	X	Y	Z	ALEx10^−2^	Cluster composition	Contributing studies (%)
C1	R Anterior Cingulate	4096	–	**0**	**42**	**−8**	3.29	90.1% Anterior Cingulate, 9.9% Medial Prefrontal Gyrus	44.0%
	L Anterior Cingulate		32	**−8**	**44**	**0**	1.77		
	L Anterior Cingulate		32	**−8**	**40**	**6**	1.71		

*ALE* Activation Likelihood Estimation, *BA* Brodmann area, *cFWE* family-wise error correction, *MNI* Montreal Neurological Institute, *SUD* substance use disorder, *R* right, *L* left.

Furthermore, the sensitivity analysis demonstrated another significant convergence cluster located within the right declive of the cerebellum and the right fusiform gyrus (Table S5, Fig. S2). However, as this cluster only became significant when the 7 studies with methodological or diagnostic caveats were excluded, and as the sensitivity analysis ALE was solely performed to assess the stability of the primary results, we refrain from further analysis or interpretation of this cluster. This is to avoid placing too much weight on an effect with an uncertain risk of being a false positive result or being of overall unreliable significance.

In contrast, the vACC cluster demonstrated clear robustness to potential publication bias. As the FSN analysis demonstrated, the vACC cluster retained statistical significance even when 30% percent of additional null studies (i.e., 7 hypothetical experiments) were introduced to the dataset. On the other hand, it did not exceed the upper FSN limit (197 hypothetical experiments), indicating that its significance is not disproportionately sustained by a small subset of influential experiments that unduly immunises this finding against extensive non-replication [26]. According to the vACC cluster’s resilience to study inclusion variability and potential publication bias all subsequent cluster characterisations and penalised regression analyses were performed using the full set (k = 23) of included studies.

### Behavioural profile and meta-analytic connectivity assessments

The vACC cluster demonstrated significant database-derived associations to exceed the multiple-comparison corrected threshold of Z = 3.0 with a variety of cognitive and affective processes related to reward/gain (Z = 6.9), reasoning (Z = 6.3), social cognition (Z = 4.3), fear (Z = 3.9), attention (Z = 3.6) and explicit memory (Z = 3.4) as indicated by the behavioural domain analysis (Fig. 1b).

Regarding its meta-analytic connectivity profile, we observed 4 clusters of significant meta-analytic connectivity. These encompass midline structures within the anterior cingulate gyri, medial frontal gyri, and posterior cingulate gyri, as well as the bilateral nuclei caudati, bilateral parahippocampi and amygdalae. Other regions of meta-analytic connectivity were more unilaterally organised demonstrating significant co-activation clusters in the left middle temporal gyrus, the right thalamus and the left inferior frontal gyrus (Fig. 1c). For a more detailed depiction of MACM metrics see Supplementary Table S6.

### Relationship of vACC contributions and study-level characteristics

**Fig. 2:**
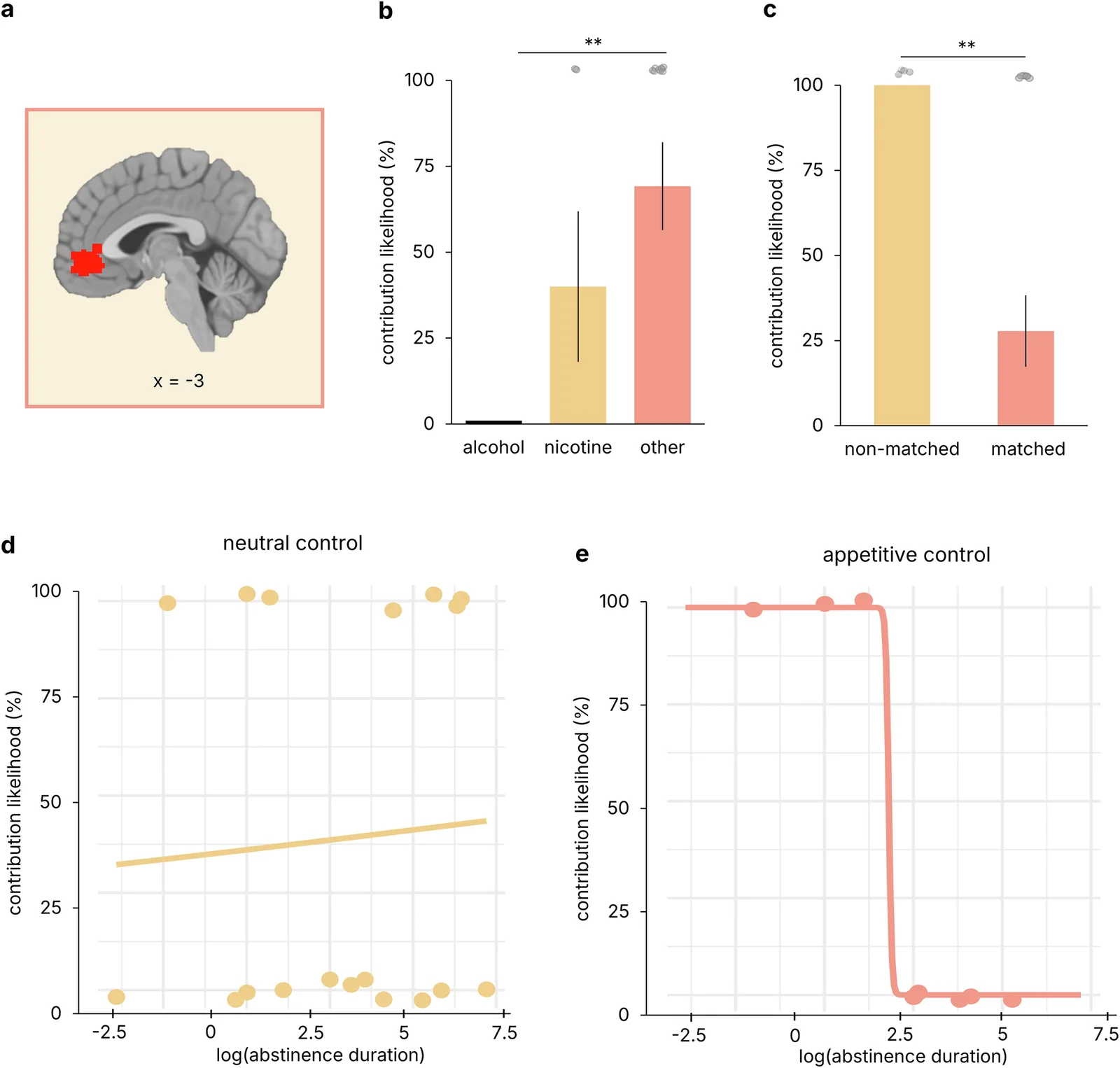
Results of the penalised logistic regression analyses linking study-level moderators to vACC cluster contributions. Displayed is the vACC as a binary mask (**a**), the effect of substance type (**b**), the effect of matching the drug condition with the control condition for human or action/tool-related content (**c**) and the interaction between abstinence duration (natural log-transformed) and control condition valence that were either neutral control conditions (**d**) or appetitive control conditions (**e**). Transforming the logarithmic values back to raw abstinence days suggests that vACC DCR is no longer evident at approximately a log-transformed abstinence duration value of 2.5, corresponding to roughly 12 days of abstinence. Note that we display the cluster contribution likelihood in percent to give a more intuitive interpretation mirroring the frequencies of observing vACC DCR. Additionally, a small jitter to the single data points that represent the included studies was introduced to increase their visibility throughout the Figure. *vACC* ventral anterior cingulate cortex.

Studies differed in their likelihood of contributing to the vACC cluster as a function of substance class (db-LRT: χ²(2) = 9.13, p = 0.010, R²_Nagelkerke_ = 0.421). Notably, no study investigating alcohol use disorder contributed to the vACC convergence, resulting in full separation. Pairwise comparisons indicated that studies investigating “other” SUDs such as heroin or methamphetamine were significantly more likely to contribute to the vACC cluster than alcohol studies (aOR = 31.66, 95%CI = 1.46–685.56, χ²(1) = 8.99, p = 0.003). Nicotine studies did not significantly differ from alcohol studies (aOR = 10.72, 95%CI = 0.40–287.97, χ²(1) = 2.66, p = 0.103) nor from the “other” substance group (aOR = 2.96, 95%CI = 0.41–21.28, χ²(1) = 1.19, p = 0.276), positioning nicotine studies as intermediate in vACC contribution likelihood (Fig. 2b).

Matching of drug and control conditions for human stimulus content also modulated vACC contribution likelihood (db-LRT: χ²(2) = 7.21, p = 0.027, R²_Nagelkerke_ = 0.344). Studies that included human stimuli in the drug condition (e.g., images depicting individuals using the drug) but not in the control condition were uniformly associated with vACC contributions, again resulting in full separation. Accordingly, studies that did not match drug and control conditions for human stimuli as opposed to matched studies demonstrated a higher likelihood of contributing to the vACC cluster (aOR = 27.03, 95%CI = 1.27–588.24, χ²(1) = 7.99, p = 0.005) (Fig. 2c). To assess whether this effect reflected the general presence of human stimuli per se rather than condition mismatch, we conducted an exploratory analysis independent of control condition matching. This analysis did not yield a significant association (db-LRT: χ²(1) = 2.08, p = 0.354), where the general inclusion of human stimuli across drug and control conditions was not associated with vACC contribution likelihood (aOR = 3.92, 95%CI = 0.68–22.76, χ²(1) = 2.52, p = 0.112). Because all studies that insufficiently matched conditions for human stimuli also failed to match action- and tool-related content, regression results for these characteristics were identical. No additional main effects of study-level characteristics significantly modulated vACC contribution likelihood.

Finally, we identified a significant interaction between abstinence duration and control condition valence (db-LRT: χ²(3) = 11.74, p = 0.008, R²_Nagelkerke_ = 0.507), indicating that the association between abstinence duration and vACC contribution likelihood depended on whether the control condition’s valence was neutral or appetitive. The interaction term suggested a decreasing likelihood of vACC contribution with increasing abstinence duration in studies employing appetitive control conditions (aOR = 0.32, 95% CI = 0.08–1.22, χ²(1) = 4.96, p = 0.026). Visual inspection of the interaction confirmed that vACC contribution likelihood was highest at shorter abstinence durations when appetitive control stimuli were used but declined with more protracted abstinence (Fig. 2d). In contrast, studies using neutral control conditions showed relatively stable contribution likelihoods across abstinence durations. No other interaction effects reached significance.

## Discussion

In this neuroimaging meta-analysis, the cue-by-group interaction yielded a single cluster in the vACC that showed above-chance convergence across studies, remained robust to potential publication bias, and withstood the sensitivity testing. A large body of research links vACC divisions to model-based computation and integration of subjective value [58, 59], as well as self-referential processing, functions that align with its role in mediating reward systems and the DMN [60]. Our database-enriched BDA and MACM analyses echo these associations, revealing a behavioural profile involving reward processing, reasoning, social cognition and explicit memory as well as significant meta-analytic co-activations with regions implicated in reward circuitry and DMN structures. Convergently, prior meta-analyses of within-subject DCR convincingly demonstrated the vACC and functionally connected circuits in neural DCR [11–13], substantiating it as a central site of neural DCR. Interestingly, our findings show that although within-subject contrasts typically yield more widespread convergence across DCR studies, only the vACC emerges as a significant site of convergence in case-control comparisons. Below, we address some questions that emerged from interpreting our ALE meta-analytic results that may support future research.

### Do stimulus matching issues for human and action/tool-related content imply bias?

In the present findings, vACC contribution likelihood was higher in studies in which drug and control cues were not matched for human features or drug-related action/tool content, suggesting a sensitivity of vACC DCR to stimulus design and selection. Because these studies lacked matching on both dimensions, it was not possible to attribute this effect to either factor in isolation. These results are in line with recent meta-analytic work of our research group on within-subject DCR [13] demonstrating that negligence of stimulus matching observed in a considerable quantity of DCR paradigms modulates the neural DCR responses. Importantly, this pattern in contrast to the within-subject DCR meta-analysis the currently observed effect is unlikely to be explained by simple perceptual mismatching, as perceptual confounds should, in principle, be evenly distributed across groups and therefore average out in between-subject designs. Instead, the findings suggest that stimulus characteristics such as the presence of human agents or drug use scenarios may selectively enhance vACC engagement in SUD samples. This interpretation is consistent with our behavioural domain analysis, which linked the vACC cluster to domains including social cognition and indicates that contextually rich or socially embedded cues may increase subjective salience in individuals with SUD. While substantial perceptual mismatches between drug and control conditions should generally be minimised for reasons concerning fMRI contrast integrity, future work may help clarify whether these effects reflect an artificial inflation of vACC responsivity associated with DCR unrelated processes involving social cognition or instead indicate a genuine amplification of vACC DCR when individuals engage with cues that may be more ecologically valid that can be utilised for more precise clinical translation. However, currently this exploratory analysis cannot establish perceptual matching as a causal explanation. Other correlated study-level characteristics may have contributed to the same pattern, and the available number of studies did not permit a reliable multivariable assessment without substantially increasing the risk of overfitting. Therefore, dedicated experimental comparisons are required to corroborate this preliminary finding.

### Does alcohol cue reactivity constitute a special case of DCR?

Interestingly, we found that the likelihood of a study contributing to the convergence in the vACC cluster differed across substance classes. Studies on typically illicit drugs showed the highest contribution rates, nicotine studies occupied an intermediate position, and alcohol studies did not contribute at all. Notably, some alcohol studies were among those using the most liberal cluster-extent thresholds, in some cases without clear information on whether these thresholds were derived from simulation-based procedures or selected a priori. Still, these studies did not contribute to the primary vACC convergence cluster, suggesting that the absence of alcohol-related contribution to vACC convergence was not driven by overly conservative thresholding practices in the source literature. Different factors potentially rather specific to alcohol DCR may dampen the detectability of vACC activation in case-control designs.

One aspect may involve neural signalling linked to variations in subjective preferences: In example, Kirsch and colleagues [61] showed that vACC responses in a sample of alcohol use disorder individuals were modulated by individual beverage preference, which also covaried with craving ratings. Alcohol cue sets often include a heterogeneous selection of alcoholic beverages that may not match participants’ personal preferences, likely reducing the salience of the cues and the overall detectability of vACC responses across a whole DCR run. This design challenge seems to be less pronounced for nicotine and other substances, where cues tend to be more generic and therefore may be more uniformly recognised as appreciable by individuals with SUD. In contrast, if drug stimuli include specific features cueing preferences, such as menthol versus non-menthol cigarettes or vaping versus combustible cigarettes, they replicate the preference signal in the vACC reported by Kirsch and colleagues [62, 63]. Extending this logic to other drug classes (e.g., opioids) may also be informative, as preferences for distinct routes of administration (e.g., smoking, snorting, or injecting heroin) are common yet rarely differentiated in cue sets or tailored to the individual. Systematically modelling route-specific preferences may also provide an important layer of personalisation that currently appears underrepresented in DCR paradigms.

A second issue may apply to differences in control group configurations: In alcohol use disorder research, control participants are frequently recreational drinkers and therefore share substantial alcohol-related learning histories with SUD participants. By contrast, studies of nicotine and other substances more often contrast SUD individuals with “never-user” controls, maximising differences in prior reward-based learning, indicating that cross-substance differences in vACC DCR may partially reflect differences in control group composition rather than substance-specific neurobiological effects. In this view, the vACC may signal not only preference but also broader reward history processes that distinguish individuals with and without prior reinforcement experience. As a result, vACC responses may not be able to cleanly separate SUD individuals from those using a drug recreationally or require a quite considerable degree of statistical power to show sufficient gradation in DCR experiments. The results may complicate interpretations of vACC DCR as a marker that is sensitive to the diagnostic category of SUD that generalises seamlessly across substance classes and instead suggests that preference-tailored considerations may be necessary to improve its translational potential.

Although further research with sufficiently powered comparisons is required, our findings point to challenges that are particularly salient for alcohol DCR designs, highlighting the need to establish standards that balance individualisation and standardisation in the selection of drug cue sets.

### Should DCR-derived evidence for ‘hyperreactive’ or ‘hijacked’ reward systems in addiction interpreted with caution?

Another central observation concerned the interaction between abstinence duration and control condition valence. While vACC responses to drug versus neutral cues appeared invariant across abstinence periods, the difference between vACC responses to drug cues compared to appetitive control cues decreased with longer abstinence. This pattern is consistent with the notion that natural rewards temporarily lose motivational relevance during withdrawal [64] and suggests that vACC-mediated responses to drug cues versus natural reward cues may track current motivational states rather than being rigidly tied to drug-related incentives under any circumstance. Accordingly, a related question concerns the close link between abstinence length and participants’ motivation to cease consumption, as this may influence whether drug cues are evaluated as rewarding or aversive and thereby shape heterogeneity in neural responses to drug cue stimulation [13, 65].

However, translational applications of DCR tend to rest on the perception that neural responses to drug cues reveal persistent pathological properties of the reward system in SUDs, where many studies refer to frameworks of hijacked or hyperreactive reward circuits in their introductions or conclusions [10, 46, 66–69] to bridge DCR to theories accenting drug cue hyper-salience, such as incentive sensitisation [8, 9]. Our findings suggest that such interpretations may require greater nuance, particularly when DCR is used to make mechanistic claims about underlying neurobiological dysfunction.

A first issue concerns the localisation of neural DCR effects. Incentive sensitisation theory predicts sensitised wanting to be expressed through cue-elicited mesolimbic reactivity, including striatal targets [9]. Across primary and supplementary analyses, we did not observe statistically significant striatal convergence (see also [13]), indicating that striatal convergence was not sufficiently discrepant from the null distribution of random spatial association to reject the null hypothesis. Instead, we observed reliable vACC convergence aligning with accounts that extend neural DCR to mesocortical contributions and that mesolimbic sites only emerged when its meta-analytic connectivity pattern is considered. These findings do not necessarily invalidate incentive sensitisation, but they raise the question of whether sensitised wanting is expressed through a striatum-centred neural correlate that can be reliably captured by common DCR imaging protocols. Furthermore, as noted in previous work [13], striatal regions are relatively small and are subject to an unfavourable signal-to-noise ratio due to their proximity to ventricular structures, which can compromise the reliable measurement of blood-oxygen-level-dependent signals [70] that survive whole-brain corrections for multiple comparisons.

However, we perceive a second interpretational constraint for inferences of hyperreactivity to be rooted in statistical group-by-cue comparisons more explicitly, where never-using controls lack any drug-related associative history. Group differences in drug cue responses may therefore reflect experiential learning rather than pathological alterations to reward circuitry. This issue becomes especially prominent in contrasts that employ natural reward control cues. Controls’ reward circuits may respond reliably to natural rewards but have no experiential basis for responding to drug cues, resulting in DCR effects being driven by asymmetrical comparisons with a statistical tendency to encourage interpretations of pathologically amplified neural responses in SUD, even when the underlying pattern may merely reflect differences in cue familiarity. This methodological speciality may differ fundamentally from case-control contrasts in other areas of translational neuroscience, such as reward processing in schizophrenia [71] or depression [72], where both groups have comparable experiential histories for the stimuli employed.

A third and, in our view, particularly consequential issue involves the choice of comparator stimuli, being an issue that is increasingly discussed in the literature [73]. We agree with previous proposals that drug versus neutral contrasts do not establish whether drug cues evoke abnormal or hyper-reactive reward signalling [74]. In contrast, tasks that compare drug cues with natural reward cues may provide information more valuable for translation by assessing the competition between salient motivational options under specific contingencies, which our results support. Notably, the pattern predicted by hijacked or hyperreactive reward accounts where drug cues exceeding natural reward responses within the SUD group, and natural reward responses being diminished relative to controls appears to be most prominent during early abstinence [51], when withdrawal-related motivational states may augment drug cue salience. However, this pattern seems less detectable during mid or long-term abstinence, where natural reward responses in vACC regions in SUD more closely resemble those of non-affected controls [17, 46], and drug cue responses do not exceed natural reward responses [17, 46, 75, 76]. Whether this abstinence-related effect reflects recovery in reward-mediating circuits [10, 77, 78] or instead pre-existing individual differences predisposing some individuals to achieve sustained abstinence remains elusive due to our meta-analysis including cross-sectional observations only. Furthermore, we can only speculate whether preferential reactivity to drug cues over natural rewards during recent abstinence transfers to states of adversity including distress, anxiety or disadvantageous socioeconomic circumstances that may predispose recurrent drug use and relapse [79] in more prolonged abstinence durations. In this regard, it may be necessary to further detangle the specificity of effects observed in the vACC or other regions for drug cue salience, positive and negative valence or general salience more broadly. In sum, our results and those of others [80, 81] suggest that clinical translations employing DCR paradigms might benefit from favouring control conditions that involve other motivationally salient comparators as opposed to motivationally inert ones.

A related consideration concerns the temporal dimension of DCR, as its clinical meaning may depend on when responses are measured along the abstinence trajectory. During early abstinence, heightened drug cue responses may be common across individuals with SUD and may primarily reflect transient motivational biases related to withdrawal relief. Because such responses may generalise broadly across SUD individuals, their utility for differentiating individual relapse risk might therefore be limited. In contrast, as abstinence lengthens and withdrawal-related states subside, persistent or re-emerging drug cue-favouring responses in reward-related circuits may become more clinically informative by reflecting a more enduring motivational pull toward drug reinforcement. Accordingly, future work might benefit from considering temporal dynamics to elucidate whether there is a sweet spot when DCR is clinically the most informative and to distinguish state-related motivational shifts from potential trait-like vulnerability markers.

## Limitations

Several limitations should be considered when interpreting the present findings. Although our primary ALE meta-analysis included 25 independent contrasts and therefore falls within the range recommended for robust ALE analyses [82], the power may still be limited for modest, less consistently reported, or spatially distributed effects. This limitation is particularly relevant because the present synthesis was based on case-control group-by-cue interaction contrasts, whose detection in the source literature may itself depend on the power of factorial fMRI designs, sample size heterogeneity, and source-study reporting thresholds. Indeed, some included studies used comparatively liberal cluster-extent thresholds, and it was not always clear whether these thresholds were derived from simulations or selected a priori. Liberal thresholding may increase the number of false-positive foci entering coordinate-based meta-analysis. However, inspection of these studies showed that they contributed relatively few foci to the ALE dataset, ranging from 1 to 7 foci per study, and did not contribute to the observed vACC convergence cluster. Thus, liberal cluster-extent thresholding among the included studies was not a driver of the observed vACC convergence.

In addition, the penalised regression models treat non-replication as reflecting genuine absence of effects rather than insufficient statistical power, a broader limitation of analysing cluster contributions of fMRI research. Relatedly, some predictors were necessarily approximated using relatively crude proxies. In example, as many studies reported abstinence only in broad ranges, we frequently relied on centroids of ranges or other approximations, which likely reduced measurement precision. Similarly, it should be noted that we chose the substance class categories primarily based on statistical practicality as there are less reports for individual substances other than alcohol and nicotine. Accordingly, our analysis cannot provide a sufficient granularity on the effects related to single substances we compiled in the “other substances” class (e.g., heroin). Clearly, further research in this regard is necessary.

Furthermore, we were unable to formally test whether the study-level moderators of cluster replicability that were examined individually shared common variance that might reflect a broader latent construct. Although we hypothesised that such shared variance could relate to contextually dynamic representations of subjective salience, incorporating multiple predictors into a single model would have substantially increased the risk of overfitting. We therefore adopted a more conservative modelling strategy.

Taken together, these limitations suggest that our meta-analytic findings may be regarded as preliminary with respect to moderators of cluster convergence. Nonetheless, they may provide a useful foundation for future hypothesis-driven work, including original studies, targeted re-analyses, and large-scale mega-analytic approaches that may overcome some of the constraints inherent to the present synthesis.

### Concluding remarks

Although our conclusions require further empirical support and may be regarded as hypothesis-generating, they offer several directions for advancing a literature that ever since encounters challenges in clinical translation and reproducibility. The clinical relevance of DCR may depend on a more precise understanding of the conditions under which it reflects transient motivational dynamics or alternatively indicates enduring vulnerability to relapse. Our findings may also encourage field wide standards in DCR task design that balance stimulus personalisation, which maximises the capture of subjective salience, with design standardisation ultimately improving comparability across studies. Progress in this area would be greatly strengthened by longitudinal research that moves beyond single time point assessments and univariate brain-behaviour statistics. From this point of view, neural DCR may be also considered within contextual frameworks of reinforcer pathology [79, 83–85] that examine how individuals with SUD balance competing motivational contingencies, and whether they may do so in ways that are more contextually adaptive than some interpretations of hyperreactive or hijacked reward-system functioning currently imply.

## Supplementary information

Supplemental Material
