# Supplementary material for: What can case-control comparisons of neural drug cue reactivity tell us about addiction? A neuroimaging meta-analysis
Source: Transl Psychiatry. 2026 Sep 17;16:501. doi: 10.1038/s41398-026-04458-9 (PMC13627667; doi:10.1038/s41398-026-04458-9)
Supplement: Supplementary file 1 [file 41398_2026_4458_MOESM1_ESM.docx]

**- Supplemental Materials -**

**What can case-control comparisons of neural drug cue reactivity tell us about addiction? A neuroimaging meta-analysis**

Maximilian Fascher^1,2,*^, Krzysztof Krasowski^1^, Markus Muehlhan^1,2^

^1^ Department of Psychology, Faculty of Human Sciences, Medical School Hamburg, Am Kaiserkai 1, 20457 Hamburg, Germany

^2^ ICAN Institute for Cognitive and Affective Neuroscience, Medical School Hamburg, Am Kaiserkai 1, 20457 Hamburg, Germany

*Corresponding author:

Maximilian Fascher

Medical School Hamburg

Am Kaiserkai 1

20457 Hamburg, Germany

Phone: +49 40 361 226 40

E-mail: maximilian.fascher@medicalschool-hamburg.de

– Table of Contents –

**1 Supplementary Methods**

1.1 Behavioural domain analysis of ALE clusters**………………………………………...3**

1.2 Meta-analytic connectivity modelling of ALE clusters**………………………………..3**

1.3 Fail-Safe-N (FSN): Assessing ALE cluster robustness towards potential publication bias**……………………………………………………………………………….4**

**2 Supplementary Tables**

**Table S1** PRISMA checklist for systematic reviews**……………………………………..6**

**Table S2** Neuroimaging meta-analysis checklist**………………………………...………9**

**Table S3** Correction of potentially misreported coordinates across included studies**……………………………………………………………………………………….11**

**Table S4** Studies omitted from the sensitivity ALE meta-analysis due to study-level caveats relevant to interpretability.**………...……………………………………………..12**

**Table S5** Sensitivity analysis ALE (p <0.001, cFWE p <0.05, 1000 permutations)**………………………………………………………………………………..13**

**Table S6** MACM results for the vACC ALE cluster (p <0.001, cFWE p <0.05, 1000 permutations)**………………………………………………………………………………..14**

**3 Supplementary Figures**

**Fig. S1** PRISMA flow chart of the systematic literature review and meta-analysis**………………………………………………………………………………………15**

**Fig. S2** Visualisation of the sensitivity analysis ALE (p <0.001, cFWE p <0.05, 1000 permutations)**……………………………………………………………………………..…16**

**Supplementary references………………………………………………………………17**

**1 Supplementary Methods**

In the following sub-sections, we describe the methods applied to the cluster of the main ALE analysis to characterise its behavioural profile, meta-analytic connectivity, and its robustness towards potential publication bias being a standard analysis pipeline in ALE meta-analyses to deepen the context of the obtained results.

1.1 Behavioural domain analysis of ALE clusters

To assess the behavioural profile of identified ALE clusters we performed a behavioural domain analysis (BDA) using the software Mango^1^ (Mango v4.1 (http://ric.uthscsa.edu/mango/; behavioral domain v3.1). Mango utilises the BrainMap database (https://brainmap.org) to calculate whether implemented experiments’ task-dependent activations significantly map onto a specified binary mask. Accordingly, the significant ALE clusters had to be transformed into Talairach reference space as this is a software requirement to be assessed for spatial covariance with the domain-associated activation sites. As larger specified masks have a higher potential to contain more raw activation counts across associated behaviours, multiple comparison correction is required. Correction for multiple comparisons was performed using a conservative Bonferroni approach with a minimum z-value threshold of z= 3.0. Containing the 5 major behaviour domains action, perception, cognition, emotion, and interoception further dividing into 59 discrete subcomponents the BDA enables a granular specification of a behavioural profile to associate with the respective cluster mask.

1.2 Meta-analytic connectivity modelling of ALE clusters

To characterise how each significant convergence cluster is involved within broader functional brain networks, we conducted meta-analytic connectivity modelling (MACM) analyses also by using data from the BrainMap database^2,3^. Using the same cluster masks as in the BDA, the defined binary masks of the ALE clusters in the Talairach reference space were entered into Sleuth (https://www.brainmap.org/sleuth/) to identify all studies in the database that report activation foci falling within that region. The experiments’ foci showing co-activation within a given mask were extracted and compiled into a text file and subjected to an ALE analysis, using the same voxel-level and cluster-level thresholds applied in the main ALE analysis (p < 0.001, cFWE p < 0.05, 1000 permutations). This procedure yielded maps indicating above-chance patterns of co-activation associated with an ALE cluster, thereby revealing how these regions participate in larger-scale functional task-positive networks. We applied several filters in Sleuth to homogenise the MACM results, including “Activations: Activations Only”, “Diagnosis: Normals”, “Imaging Modality: fMRI”, “Context: Normal Mapping” and “Subjects: Size >9”.

1.3 Fail-Safe-N: Assessing ALE cluster robustness towards potential publication bias

We used the Fail-Safe-N (FSN) method^4^ to assess the robustness of ALE-derived clusters against potential publication bias – commonly referred to as the “file-drawer problem”. Since ALE only includes significant peak coordinates, null findings cannot be incorporated into the meta-analysis, potentially introducing a one-sided confirmation bias and overestimating the credibility of the evidence. Simulations suggest that 6-30% of neuroimaging studies remain unpublished due to non-significant results^5^. To address this, Acar et al.^4^ developed an ALE-specific FSN method that estimates the number of hypothetical null studies a cluster can withstand before losing statistical significance. The ideal FSN represents a number that lies between two boundaries: a lower bound, reflecting the minimum number of added null studies a cluster must withstand to retain statistical significance, and an upper bound, beyond which significance may be overly dependent on a small number of disproportionately influential studies. Although the choice of these bounds is ultimately at the researcher’s discretion, we defined the lower boundary as requiring clusters to survive an additional 30% of null studies, adopting this conservative threshold to accommodate the prevalence of publication bias. We calculated this as “(number of included studies)×0.3”, rounding up to the next whole number. For the upper boundary, Acar et al. argue that if only 5-10% of studies drive a significant ALE cluster, it may reflect an ALE cluster to be driven by small yet disproportionately influential studies making it immune to repeated and excessive non-replication. We conservatively set the upper boundary at 5%, computed using the formula “((number of studies contributing to the cluster/0.05) – (number of included studies))”. In summary, an ALE cluster was considered robust but not inflated, if it remained significant after adding 30% null studies (in our case n= 7 additional null studies) and lost significance when the original studies represented only 5% (in our case n= 197 additional null studies) of the total FSN-adjusted sample, respectively.

Following Acar et al.’s procedure, we used their R code provided along their paper (https://github.com/NeuroStat/GenerateNull) in R statistics v4.4.3 to generate artificial null studies (i.e. noise studies) that are similar in sample size and number of foci to the actual included studies, but whose peaks were randomly distributed within the whole brain mask. These were added to the original ALE input serving as artificial noise, and the meta-analysis was rerun. To reduce computational demands and this procedure is based on manual iterations, we evaluated whether each cluster’s FSN fell within this boundary range rather than calculating the exact FSN. All FSN ALE computations were thresholded at voxel-wise p < 0.001 and cFWE cluster-level p < 0.05 with 1000 permutations as for the main analysis.

**2 Supplementary Tables**

| **Table S1.** PRISMA checklist for systematic literature reviews and meta-analyses^6^. Note that for fMRI meta-analysis, not all criteria proved to be feasible. | | | |
| --- | --- | --- | --- |
| **Section and Topic** | **Item #** | **Checklist item** | **Location where the item is reported** |
| **TITLE** | | |  |
| Title | 1 | Identify the report as a systematic review. | Title, Introduction |
| **ABSTRACT** | | |  |
| Abstract | 2 | See the PRISMA 2020 for Abstracts checklist. | Abstract |
| **INTRODUCTION** | | |  |
| Rationale | 3 | Describe the rationale for the review in the context of existing knowledge. | Introduction |
| Objectives | 4 | Provide an explicit statement of the objective(s) or question(s) the review addresses. | Introduction |
| **METHODS** | | |  |
| Eligibility criteria | 5 | Specify the inclusion and exclusion criteria for the review and how studies were grouped for the syntheses. | 2 Methods, first paragraph |
| Information sources | 6 | Specify all databases, registers, websites, organisations, reference lists and other sources searched or consulted to identify studies. Specify the date when each source was last searched or consulted. | 2 Methods, first paragraph |
| Search strategy | 7 | Present the full search strategies for all databases, registers and websites, including any filters and limits used. | 2 Methods, first paragraph |
| Selection process | 8 | Specify the methods used to decide whether a study met the inclusion criteria of the review, including how many reviewers screened each record and each report retrieved, whether they worked independently, and, if applicable, details of automation tools used in the process. | 2 Methods, first paragraph |
| Data collection process | 9 | Specify the methods used to collect data from reports, including how many reviewers collected data from each report, whether they worked independently, any processes for obtaining or confirming data from study investigators, and, if applicable, details of automation tools used in the process. | 2 Methods, first paragraph |
| Data items | 10a | List and define all outcomes for which data were sought. Specify whether all results that were compatible with each outcome domain in each study were sought (e.g. for all measures, time points, analyses), and if not, the methods used to decide which results to collect. | 2 Methods, first paragraph |
|  | 10b | List and define all other variables for which data were sought (e.g. participant and intervention characteristics, funding sources). Describe any assumptions made about any missing or unclear information. | 2 Methods, first paragraph |
| Study risk of bias assessment | 11 | Specify the methods used to assess risk of bias in the included studies, including details of the tool(s) used, how many reviewers assessed each study and whether they worked independently, and if applicable, details of automation tools used in the process. | 2 Methods, first paragraph |
| Effect measures | 12 | Specify for each outcome the effect measure(s) (e.g. risk ratio, mean difference) used in the synthesis or presentation of results. | 2 Methods,  “ALE” paragraph |
| Synthesis methods | 13a | Describe the processes used to decide which studies were eligible for each synthesis (e.g. tabulating the study intervention characteristics and comparing against the planned groups for each synthesis (item #5)). | not feasible |
|  | 13b | Describe any methods required to prepare the data for presentation or synthesis, such as handling of missing summary statistics, or data conversions. | 2 Methods, “ALE” paragraph |
|  | 13c | Describe any methods used to tabulate or visually display results of individual studies and syntheses. | not feasible |
|  | 13d | Describe any methods used to synthesize results and provide a rationale for the choice(s). If meta-analysis was performed, describe the model(s), method(s) to identify the presence and extent of statistical heterogeneity, and software package(s) used. | 2 Methods, “ALE” paragraph |
|  | 13e | Describe any methods used to explore possible causes of heterogeneity among study results (e.g. subgroup analysis, meta-regression). | 2 Methods, “Linking ALE cluster contributions to clinical variables ” paragraph |
|  | 13f | Describe any sensitivity analyses conducted to assess robustness of the synthesized results. | 3 Results, first paragraph, Supplementary Table S5 |
| Reporting bias assessment | 14 | Describe any methods used to assess risk of bias due to missing results in a synthesis (arising from reporting biases). | Supplementary methods, “Fail-Safe-N: Assessing ALE cluster robustness towards potential publication bias” |
| Certainty assessment | 15 | Describe any methods used to assess certainty (or confidence) in the body of evidence for an outcome. | Not applicable |
| **RESULTS** | | |  |
| Study selection | 16a | Describe the results of the search and selection process, from the number of records identified in the search to the number of studies included in the review, ideally using a flow diagram. | 3 Results first paragraph, Supplementary Figure S1 |
|  | 16b | Cite studies that might appear to meet the inclusion criteria, but which were excluded, and explain why they were excluded. | Not applicable |
| Study characteristics | 17 | Cite each included study and present its characteristics. | Table 1 |
| Risk of bias in studies | 18 | Present assessments of risk of bias for each included study. | Not applicable |
| Results of individual studies | 19 | For all outcomes, present, for each study: (a) summary statistics for each group (where appropriate) and (b) an effect estimate and its precision (e.g. confidence/credible interval), ideally using structured tables or plots. | Not applicable |
| Results of syntheses | 20a | For each synthesis, briefly summarise the characteristics and risk of bias among contributing studies. | Not applicable |
|  | 20b | Present results of all statistical syntheses conducted. If meta-analysis was done, present for each the summary estimate and its precision (e.g. confidence/credible interval) and measures of statistical heterogeneity. If comparing groups, describe the direction of the effect. | 3 Results, “ALE meta-analysis results and robustness assessments |
|  | 20c | Present results of all investigations of possible causes of heterogeneity among study results. | 3 Results, “Relationship of vACC contributions and study-level characteristics” |
|  | 20d | Present results of all sensitivity analyses conducted to assess the robustness of the synthesized results. | 3 Results, “ALE meta-analysis results and robustness assessments” |
| Reporting biases | 21 | Present assessments of risk of bias due to missing results (arising from reporting biases) for each synthesis assessed. | Not applicable |
| Certainty of evidence | 22 | Present assessments of certainty (or confidence) in the body of evidence for each outcome assessed. | Not applicable |
| **DISCUSSION** | | |  |
| Discussion | 23a | Provide a general interpretation of the results in the context of other evidence. | 4 Discussion |
|  | 23b | Discuss any limitations of the evidence included in the review. | 4 Discussion |
|  | 23c | Discuss any limitations of the review processes used. | 4 Discussion |
|  | 23d | Discuss implications of the results for practice, policy, and future research. | 4 Discussion |
| **OTHER INFORMATION** | | |  |
| Registration and protocol | 24a | Provide registration information for the review, including register name and registration number, or state that the review was not registered. | 2 Methods, first paragraph |
|  | 24b | Indicate where the review protocol can be accessed, or state that a protocol was not prepared. | not applicable |
|  | 24c | Describe and explain any amendments to information provided at registration or in the protocol. | Not applicable |
| Support | 25 | Describe sources of financial or non-financial support for the review, and the role of the funders or sponsors in the review. | “Funding” |
| Competing interests | 26 | Declare any competing interests of review authors. | „Conflict of interest“‘ |
| Availability of data, code and other materials | 27 | Report which of the following are publicly available and where they can be found: template data collection forms; data extracted from included studies; data used for all analyses; analytic code; any other materials used in the review. | Not applicable |

| **Table S2.** Neuroimaging meta-analyses checklist provided by Müller et al.^7^ | |
| --- | --- |
| **The research question is specifically defined** | **✓** Yes, we investigated the neural underpinnings of case-control contrasts in neural drug cue reactivity in substance use disorders and factors modulating them  *🡪 1 Introduction* |
| **The literature search was systematic** | **✓** The following search string has been applied:  (“cue reactivity” OR “cue-reactivity” OR “cue exposure” OR “cue-exposure” OR “cue-elicited craving” OR “drug cue reactivity” OR “drug cue-reactivity” OR “drug cue exposure” OR “drug-cue exposure” OR “drug cue-elicited craving”) AND (“functional magnetic resonance imaging” OR “fMRI” OR “functional MRI”) AND (“substance use disorder” OR “addiction” OR “drug abuse” OR “drug dependence” OR “alcohol use disorder” OR “alcoholism” OR “alcohol” OR “drinker*” OR “cocaine” OR “crack” OR “methamphetamine” OR “crystal meth” OR “amphetamine” OR “stimulant*” OR “tobacco” OR “smok*” OR “cannabis” OR “THC” OR “opioid*” OR “heroin” OR “morphine” OR “codeine” OR “ketamine” OR “polytox*” OR “polysubstance” OR “substance use*”)  Databases:  PsycINFO, PsycARTICLES, Medline Complete, CINAHL Complete and Psychology, Behavioral Sciences Collection, and PubMed  *🡪 2 Methods: “Systematic literature search”* |
| **Detailed inclusion and exclusion criteria are included** | **✓** We formulated in-/exclusion criteria a priori in a preregistration based on state-of-the-art recommendations for neuroimaging meta-analyses  *🡪 2 Methods: “Systematic literature search”* |
| **Sample overlap was taken into account** | **✓** We suspected some sample overlap, which when detected led to exclusion of the lesser powered study to minimise noise and redundancy.  🡪 2 *Methods “Systematic literature search”* |
| **All experiments use the same search coverage (state how brain coverage is assessed and how small volume corrections and conjunctions are taken into account)** | **✓** All experiments must contain whole brain analyses or almost whole-brain coverage. Whole brain coverage was assessed by scanning parameters reported in original studies and compared to average brain measures provided by Müller et al. (2018). Almost whole-brain coverage was considered in sensitivity analysis  🡪 *2 Methods: “Systematic literature search and inclusion criteria”* |
| **Studies are converted to a common**  **reference space** | **✓** Coordinates reported in Talairach reference space were transformed into MNI space by applying the Lancaster transform icbm2tal implemented in GingerALE software.  🡪 *2 Methods: “Activation Likelihood Estimation and consecutive cluster assessments”* |
| **Data extraction have been conducted by two investigators (ideal case) or double checked by the same investigator (state how doublechecking was performed)** | **✓** As follows:  Researcher MFconducted the systematic literature search, MF and KK checked inclusion criteria independently and discussed study inclusion. Researcher MM was consulted when necessary. MF extracted and double-checked coordinates and extracted sample characteristics. MM supervised the data  *🡪 2 Methods, “Systematic literature search and inclusion criteria”* |
| **The paper includes a table with at least the references, basic study description (e.g., for fMRI tasks: stimuli), contrasts and basic sample descriptions (e.g., size, mean age and gender distribution, specific characteristics) of the included studies, source of information (e.g., contact with authors), reference space** | **✓** Yes  🡪 *“Table 1”* |
| **The study protocol was previously registered and all analyses planned beforehand, including the methods and parameters used for inference, correction for multiple testing, etc.** | **✓** We pre-registered our systematic review and meta-analysis on PROSPERO before starting the systematic literature search as part of a previous meta-analysis. All post-hoc analysis decisions were prompted by a previous ALE meta-analysis of our research group and were dependent on results that could not be anticipated without knowing the actual data structure.  🡪 *2 Methods: “Linking ALE cluster contributions to clinical variables ”* |
| **The meta-analysis includes diagnostics** | **✓** Diagnostics of the significant ALE-derived clusters are provided in Table 2, Table S5 and were part of our paragraph “Linking ALE cluster contributions to clinical variables” paragraph  🡪 *2 Methods,* *3 Results* |

| **Table S3.** Potentially misreported foci from included studies were identified after a gross plausibility check with anatomical labelling and fMRI maps reported by the respective papers. The adjusted coordinates have been entered into the ALE meta-analyses. The red colour indicates the dimension that has been suspected to be misreported and the green colour shows how we adjusted the respective coordinates. | | |
| --- | --- | --- |
| Publication | Original coordinate(s) (x,y,z) | Adjusted coordinate(s) (x,y,z) |
| Brumback et al. (2015)^8^ | 10.5, 76.5, -21.5  −31.5, 49.5, −18.5  10.5, 28.5, −3.5 | -10.5, -76.5, -21.5  31.5 -49.5 -18.5  -10.5 -28.5 -3.5 |
| Dakhili et al. (2022)^9^ | -4 -56 0 | -4 56 0 |

| **Table S4.** Studies omitted from the sensitivity ALE meta-analysis due to study-level caveats relevant to interpretability. | |
| --- | --- |
| **Study** | **Reason(s) to be left out in the sensitivity analysis** |
| Brumback et al. (2015)^8^ | No formal SUD diagnosis. However, the behavioural data indicate that participants largely meet SUD-criteria |
| Filbey et al. (2016)^10^ | Control group encompasses individuals with alcohol use disorder. However, due to the drug cue reactivity paradigm targeting responses towards cannabis cues, this study has been included in sensitivity analyses. |
| Huang et al. (2018)^11^ | The section on design and statistical analysis is not specific enough. The design apparently introduces carry-over effects between blocks of different conditions. |
| Ihssen et al. (2011)^12^ | No formal SUD diagnosis. However, the behavioural data indicate that participants largely meet SUD-criteria. |
| Liao et al. (2018)^13^ | It remains unclear whether the baseline condition was modelled explicitly in the first-level analysis. Cue conditions were very long with each lasting 2 minutes making them susceptible to noise of different sources. |
| MacNiven et al. (2018)^14^ | Low-level perceptual features of cue classes are not well matched. Furthermore, the modelling of the cue reactivity condition trials lasting 8 seconds encompasses an anticipatory response induced by reliably presenting shapes, the actual visual drug cue and a rating period. Therefore, the BOLD signals acquired during each condition may not capture only neural responsiveness to different cue types. |
| Zhang et al. (2011)^15^ | Apparently almost whole-brain coverage. Spatial resolution was very low. |

| **Table S5.** Sensitivity analysis ALE for the contrast drug > control in SUD > control participants surpassing a cluster-forming threshold of p <0.001, cluster-corrected at cFWE p <0.05 with 1000 permutations | | | | | | | | | |
| --- | --- | --- | --- | --- | --- | --- | --- | --- | --- |
|  |  | | | MNI (peak voxel) | | |  | | |
| # | Anatomical peak region | mm³ | BA | X | Y | Z | ALEx10^-2^ | Cluster composition | Contributing studies (%) |
| *SUD > controls x drug > control (k = 18 contrasts)* | | | | | | | | | |
| C1 | R Anterior Cingulate | 1,992 | - | **0** | **44** | **-8** | 1.96 | 77.5% Anterior Cingulate, 22.5% Medial Frontal Gyrus | 44.4% |
|  | L Anterior Cingulate |  | 32 | **-8** | **44** | **0** | 1.55 |  |  |
|  | L Medial Frontal Gyrus |  | 10 | **-10** | **54** | **-4** | 1.17 |  |  |
|  | L Anterior Cingulate |  | 24 | **-4** | **36** | **4** | 1.15 |  |  |
| C2 | R Fusiform Gyrus | 680 | 19 | **34** | **-82** | **-12** | 1.65 | 72.5% Declive, 25.5% Fusiform Gyrus | 22.2% |
|  | R Declive |  | - | **40** | **-74** | **-20** | 1.15 |  |  |
| Abbreviations. *ALE* Activation Likelihood Estimation, *BA* Brodmann area, *C* cluster, *cFWE* family-wise error correction, *MNI* Montreal Neurological Institute, *SUD* substance use disorder, *R* right, *L* left | | | | | | | | | |

| **Table S6.** MACM results for the vACC ALE cluster (p <0.001, cFWE p <0.05, 1000 permutations) | | | | | |
| --- | --- | --- | --- | --- | --- |
| **Cluster #** | **x** | **y** | **z** | **Peak ALE** | **Label (Nearest Gray matter)** |
| 1 | -2 | 48 | -8 | 0.3103985 | Left Anterior Cingulate |
|  | -22 | -10 | -16 | 0.09905065 | Left Amygdala |
|  | -10 | 12 | -6 | 0.092218794 | Left Caudate Head |
|  | -4 | 56 | 20 | 0.08238655 | Left Medial Frontal Gyrus |
|  | 10 | 12 | -8 | 0.07883222 | Right Caudate Head |
|  | -28 | 12 | -20 | 0.051839124 | Left Inferior Frontal Gyrus |
|  | -24 | -36 | -14 | 0.05036816 | Left Parahippocampal Gyrus |
|  | -30 | -30 | -20 | 0.043264933 | Left Parahippocampal Gyrus |
| 2 | -4 | -52 | 30 | 0.1176694 | Left Posterior Cingulate |
|  | -6 | -56 | 20 | 0.10651982 | Left Posterior Cingulate |
|  | 0 | -18 | 40 | 0.059185326 | Left Cingulate |
| 3 | 24 | -8 | -18 | 0.1069926 | Right Amygdala |
|  | 26 | -22 | -14 | 0.06819248 | Right Parahippocampus |
|  | 20 | -26 | -10 | 0.051455136 | Right Thalamus (Medial Geniculum Body) |
| 4 | -44 | -76 | 34 | 0.082775585 | Left Middle Temporal Gyrus |
|  | -50 | -64 | 30 | 0.06510237 | Left Middle Temporal Gyrus |
| Abbreviations. *ALE* Activation Likelihood Estimation, *cFWE* family-wise error correction, *MACM* meta-analytic connectivity modelling, *vACC* ventral anterior cingulate cortex | | | | | |

**3 Supplementary Figures**

**Fig. S1** PRISMA flow chart (Page et al.^6^)

**Fig. S2** Visual depiction of the sensitivity ALE analysis (p < 0.001, cFWE p < 0.05, 1000 permutations). The left panel shows the vACC cluster that has been confirmed to survive the exclusion of experiments with methodological caveats. The right panel displays the cluster peaking in the cerebellum and fusiform gyrus that did not maintain its significance in the main ALE result. *ALE* Activation Likelihood Estimation, *cFWE* family-wise error correction, *vACC* ventral anterior cingulate

**Supplementary References**

1	Lancaster JL, Laird AR, Eickhoff SB, Martinez MJ, Fox PM, Fox PT. Automated regional behavioral analysis for human brain images. *Front Neuroinform* 2012; **6**. doi:10.3389/fninf.2012.00023.

2	Eickhoff SB, Bzdok D, Laird AR, Roski C, Caspers S, Zilles K *et al.* Co-activation patterns distinguish cortical modules, their connectivity and functional differentiation. *NeuroImage* 2011; **57**: 938–949.

3	Langner R, Rottschy C, Laird AR, Fox PT, Eickhoff SB. Meta-analytic connectivity modeling revisited: Controlling for activation base rates. *NeuroImage* 2014; **99**: 559–570.

4	Acar F, Seurinck R, Eickhoff SB, Moerkerke B. Assessing robustness against potential publication bias in Activation Likelihood Estimation (ALE) meta-analyses for fMRI. *PLoS ONE* 2018; **13**: e0208177.

5	Samartsidis P, Montagna S, Laird AR, Fox PT, Johnson TD, Nichols TE. Estimating the prevalence of missing experiments in a neuroimaging meta‐analysis. *Research Synthesis Methods* 2020; **11**: 866–883.

6	Page MJ, McKenzie JE, Bossuyt PM, Boutron I, Hoffmann TC, Mulrow CD *et al.* The PRISMA 2020 statement: An updated guideline for reporting systematic reviews. *International Journal of Surgery* 2021; **88**: 105906.

7	Müller VI, Cieslik EC, Laird AR, Fox PT, Radua J, Mataix-Cols D *et al.* Ten simple rules for neuroimaging meta-analysis. *Neuroscience & Biobehavioral Reviews* 2018; **84**: 151–161.

8	Brumback T, Squeglia LM, Jacobus J, Pulido C, Tapert SF, Brown SA. Adolescent heavy drinkers’ amplified brain responses to alcohol cues decrease over one month of abstinence. *Addict Behav* 2015; **46**: 45–52.

9	Dakhili A, Sangchooli A, Jafakesh S, Zare-Bidoky M, Soleimani G, Batouli SAH *et al.* Cue-induced craving and negative emotion disrupt response inhibition in methamphetamine use disorder: Behavioral and fMRI results from a mixed Go/No-Go task. *Drug Alcohol Depend* 2022; **233**: 109353.

10	Filbey FM, Dunlop J, Ketcherside A, Baine J, Rhinehardt T, Kuhn B *et al.* fMRI study of neural sensitization to hedonic stimuli in long-term, daily cannabis users. *Hum Brain Mapp* 2016; **37**: 3431–3443.

11	Huang S, Zhang Z, Dai Y, Zhang C, Yang C, Fan L *et al.* Craving Responses to Methamphetamine and Sexual Visual Cues in Individuals With Methamphetamine Use Disorder After Long-Term Drug Rehabilitation. *Front Psychiatry* 2018; **9**: 145.

12	Ihssen N, Cox WM, Wiggett A, Fadardi JS, Linden DEJ. Differentiating heavy from light drinkers by neural responses to visual alcohol cues and other motivational stimuli. *Cerebral Cortex* 2011; **21**: 1408–1415.

13	Liao Y, Johnson M, Qi C, Wu Q, Xie A, Liu J *et al.* Cue-Induced Brain Activation in Chronic Ketamine-Dependent Subjects, Cigarette Smokers, and Healthy Controls: A Task Functional Magnetic Resonance Imaging Study. *Front Psychiatry* 2018; **9**: 88.

14	MacNiven KH, Jensen ELS, Borg N, Padula CB, Humphreys K, Knutson B. Association of Neural Responses to Drug Cues With Subsequent Relapse to Stimulant Use. *JAMA Netw Open* 2018; **1**: e186466.

15	Zhang X, Salmeron BJ, Ross TJ, Gu H, Geng X, Yang Y *et al.* Anatomical differences and network characteristics underlying smoking cue reactivity. *NeuroImage* 2011; **54**: 131–141.
